# Supplementary material for: Ferricyanide photo-aquation pathway revealed by combined femtosecond Kβ main line and valence-to-core x-ray emission spectroscopy
Source: Nat Commun. 2023 May 5;14:2443. doi: 10.1038/s41467-023-37922-x (PMC10163258; doi:10.1038/s41467-023-37922-x)
Supplement: Supplementary file 1 — Supplementary Information [file 41467_2023_37922_MOESM1_ESM.pdf]

Supplementary Information

Ferricyanide photo-aquation pathway revealed by combined femtosecond Kβ main line and valence-to-core x-ray emission spectroscopy

Marco Reinhard[1*], Alessandro Gallo[1], Meiyuan Guo[1], Angel T. Garcia-Esparza[1], Elisa Biasin[2], Muhammad Qureshi[1], Alexander Britz[1], Kathryn Ledbetter[3^], Kristjan Kunnus[1], Clemens Weninger[1#], Tim van Driel[1], Joseph Robinson[1], James M. Glownia[1], Kelly J. Gaffney[1], Thomas Kroll[1], Tsu-Chien Weng[4], Roberto Alonso-Mori[1*], Dimosthenis Sokaras[1*]

[1]SLAC National Accelerator Laboratory, Menlo Park, California 94025, USA

[2]Physical Sciences Division, Pacific Northwest National Laboratory, Richland, Washington 99352, USA

[3]Department of Physics, Stanford University, Stanford, California 94305, USA

[4]School of Physical Science and Technology, ShanghaiTech University, Shanghai 201210, China

Present addresses:

[^]Department of Physics, Harvard University, Cambridge, MA 02138

[#]MAX IV Laboratory, Lund University, 221 00 Lund, Sweden

[*]Corresponding authors:

E-mail: marcor@slac.stanford.edu, robertoa@slac.stanford.edu, dsokaras@slac.stanford.edu

Supplementary Note 1: Spectral evolution of the Fe Kβ main line XES difference spectra in the 1.5 – 40 ps range

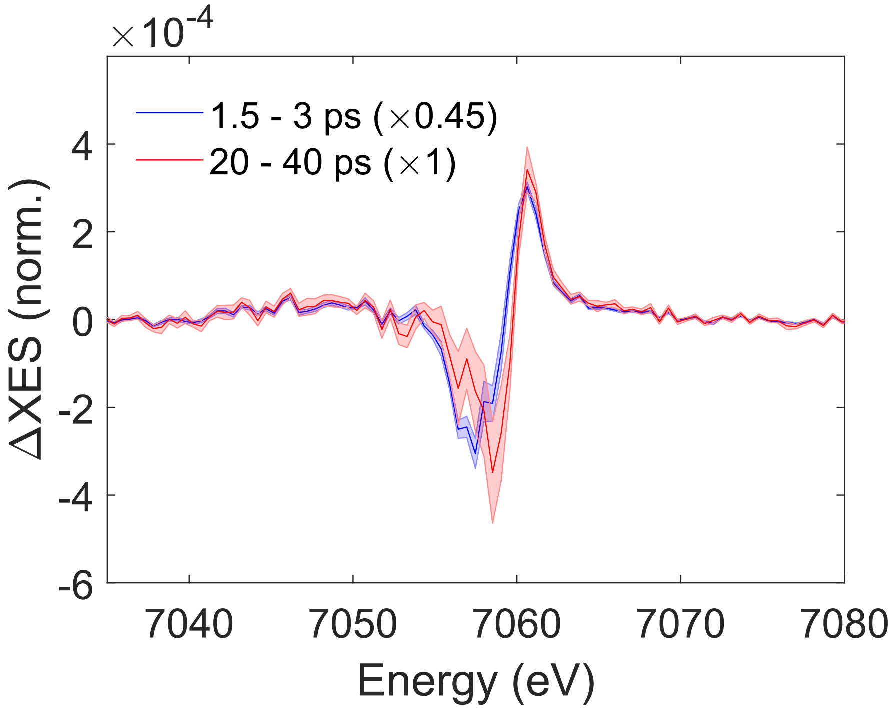

Supplementary Figure 1: Comparison between the noise-filtered Fe Kβ main line x-ray emission difference spectra averaged in the ranges 1.5 – 3 ps and 20 – 40 ps and spectrally binned. Noise-filtering was performed using a singular value decomposition and subsequent data reconstruction with five components. Uncertainties were estimated at each energy point by first calculating the standard deviation within a time bin when all difference spectra are rescaled to the bin-averaged summed difference signal magnitude. These standard deviations were then further propagated when binning spectrally.

Supplementary Note 2: Combined Fe Kβ main line and VtC XES difference spectra in the 0.1 – 0.3 ps range

Supplementary Figures 2a-b show a comparison of the Fe Kβ main line and VtC XES difference spectra averaged in the 0.1 – 0.3 ps range with the scaled difference of the $^{1}[Fe^{II}(CN)_6]^{4-}$ and $^{2}[Fe^{III}(CN)_6]^{3-}$ ground state spectra (LMCT model). In Supplementary Figures 2c-d, the residuals are compared with the XES difference averaged in the 1.5 – 3 ps range. The similarity indicates that the predominant species of the 1.5 – 3 ps range also contributes in the 0.1 – 0.3 ps range. Shaded areas shown in Supplementary Figure 2 reflect the standard deviation within a time bin when all difference spectra are rescaled to the summed difference signal magnitude of the bin-averaged spectrum.

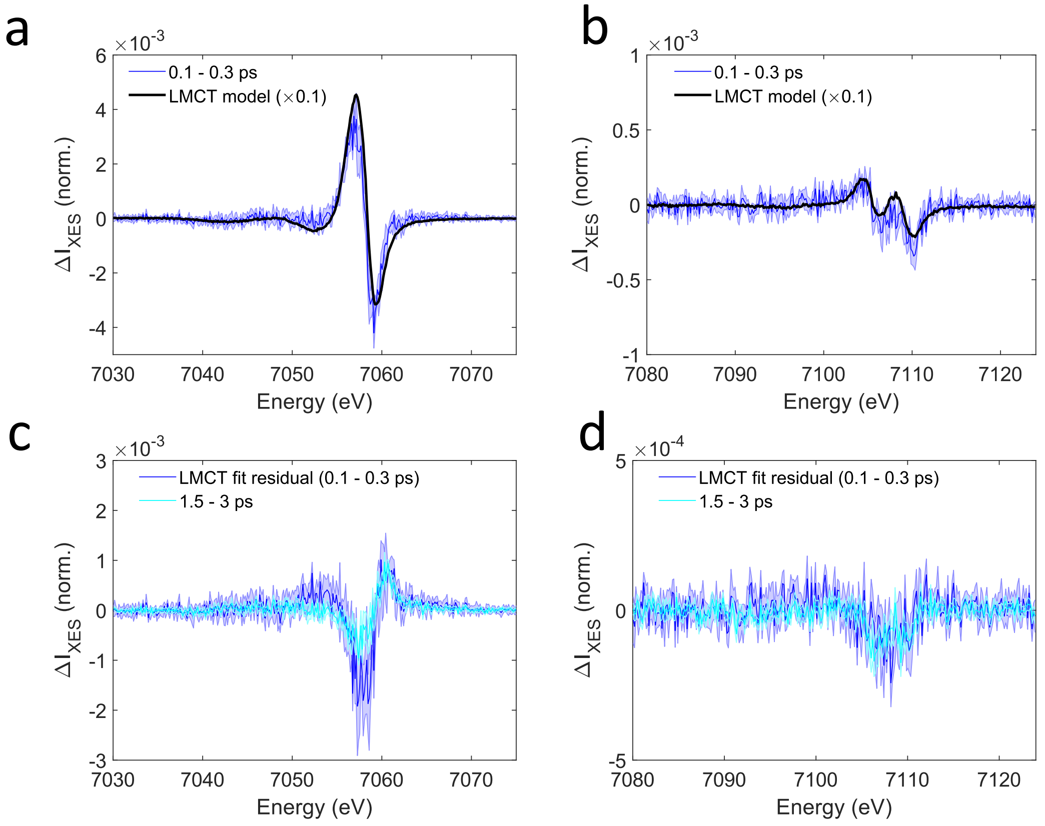

Supplementary Figure 2: (a) and (b) show the Kβ main line and VtC difference spectra in the 0.1 – 0.3 ps range together with the scaled LMCT model difference spectrum described in the main text. A common scaling factor (×0.1) is used based on Figures 2a and 3a. (c) and (d) show the residuals of the curves in (a) and (b) together with the difference spectra averaged in the 1.5 – 3 ps range. Shaded areas reflect the standard deviation within a time bin when all difference spectra are rescaled to the bin-averaged summed difference signal magnitude.

Supplementary Note 3: Population analysis of the Fe Kβ main line XES difference spectra

The time dependent populations N_{LMCT} and N_{S2} resulting from the Fe Kβ main line XES analysis were fitted using a sum of exponential functions multiplied with a Heaviside step function H and convoluted with a Gaussian instrument response function:

$$F(\sigma, t_0, a_1, a_2, a_3, \tau_1, \tau_2, \tau_3, t) \frac{1}{\sqrt{2\pi}\sigma} e^{-\frac{(t-t_0)^2}{2\sigma^2}} \otimes \sum_{i=1}^{3} a_i \cdot H(t-t_0) \cdot e^{-(t-t_0)/\tau_i} \qquad (1)$$

The fit parameters are the width of the instrument response function σ ($\mathrm{IRF\ FWHM} = 2.355 \cdot \sigma$), time zero position t_0, the amplitudes a_i, and timescales τ_i. We have compared different models to fit N_{LMCT} / N_{S2} in Supplementary Table 1 / 2, respectively. For N_{LMCT}, we did not fit a signal rise time because population of the $^2\mathrm{T}_{2u}$ LMCT ES is a direct consequence of the photo-excitation process and the rise time of N_{LMCT} should therefore be IRF-limited. For models *A* - *C* in Supplementary Table 1, the fit was performed using equation 1 with a mono-exponential decay ($a_2 = a_3 = 0$) and the IRF FWHM was fixed to 75/100/150 fs. Model *D* uses a mono-exponential decay and a floating IRF FWHM and model *E* additionally uses a long-lived component fixed to $\tau_3 = 1000\ ps$. The fit associated with model *D* is shown in Figure 2b because the coefficient of determination (R^2) is higher than for *A* – *C* and comparable with the more complex model *E*.

	A	*B*	*C*	*D*	*E*
IRF FWHM (fs)	75 (fixed)	100 (fixed)	150 (fixed)	222 ± 29	226 ± 29
$N_{LMCT}(t = 0)$ (-)	0.13 ± 0.01	0.14 ± 0.01	0.16 ± 0.01	0.19 ± 0.02	0.19 ± 0.02
τ_1 (fs)	349 ± 46	328 ± 38	294 ± 27	253 ± 27	239 ± 32
τ_2 (ps)	-	-	-	-	-
τ_3 (ps)	-	-	-	-	1000 (fixed)
R^2 (-)	0.929	0.947	0.970	0.979	0.979

Supplementary Table 1: Summary for fitting the time dependent LMCT population N_{LMCT} using equation 1 with up to two exponential decay constants. *A* - *C*: Effect of varying the IRF FWHM parameter. *D*: Fitting N_{LMCT} with one decay constant τ_1 and floating IRF FWHM parameter. *E*: Fitting N_{LMCT} with one fast decay constant τ_1, floating IRF FWHM parameter and a long-lived component fixed to τ_3 = 1000 ps.

To fit N_{S2}, models *A* – *C* in Supplementary Table 2 use equation 1 with one varying exponential decay constant τ_2, a long-lived component fixed to $\tau_3 = 1000\ ps$ and an IRF FWHM fixed to 75/100/150 fs. For model *D*, we fix the IRF FWHM to 222 fs, the value determined from fitting N_{LMCT} with model *D* in Supplementary Table 1. The large uncertainties associated with the extracted time dependent population N_{S2} do not allow a more accurate determination of the time resolution or resolving a signal rise time. The fit associated with model *D* is shown in Figure 2c.

	A	*B*	*C*	*D*
IRF FWHM (fs)	75 (fixed)	100 (fixed)	150 (fixed)	222 (fixed)
τ_2 (ps)	3.6 ± 2.1	3.5 ± 2.0	3.0 ± 1.6	2.9 ± 1.5
τ_3 (ps)	1000 (fixed)	1000 (fixed)	1000 (fixed)	1000 (fixed)
R^2 (-)	0.950	0.951	0.953	0.955

Supplementary Table 2: Summary for fitting the time dependent population N_{S2} using equation 1 with two exponential decay constants. The longer decay constant is fixed to τ_3 = 1000 ps since no decay is observed within the ~40 ps measurement window. *A* - *C*: Effect of varying the IRF FWHM parameter. *D*: Fixing the IRF FWHM parameter to the fitted value from the LMCT ES population fit (model *D* in Supplementary Table 1).

The estimated IRF FWHM of ~222 fs requires a few comments. We utilized ~50 fs optical excitation pulses, ~40 fs x-ray pulses and a 50 µm aqueous sample solution jet that smears out the time resolution due to a group velocity mismatch of ~1.5 fs/µm. The estimated time resolution is therefore IRF FWHM ~$\sqrt{50\ fs^2 + 40\ fs^2 + 75\ fs^2} \approx 100\ fs$, much shorter than the fitted value IRF FWHM ~222 fs. The origin of this difference remains unclear. However, it may in part arise from thermal drifts and vibrational effects between the sample excitation and time tool optical laser paths. Such effects may further smear out the time resolution as the relatively small VtC XES difference signal magnitudes required an extended data collection period (9 - 10 hours). Given the limited signal-to-noise ratio of individual scans or subsets of scans, we were unable to clearly resolve and correct such potential variations in time zero.

Supplementary Note 4: Analysis of the valence-to-core XES difference spectra and DFT calculations

To unambiguously verify the presence of two spectral components in the XES data, we have performed a singular value decomposition. The reconstruction of the original data shown in Supplementary Figure 3a clearly requires two components shown in Supplementary Figure 3b. Supplementary Figure 3c shows the two components (scaled) in the VtC region.

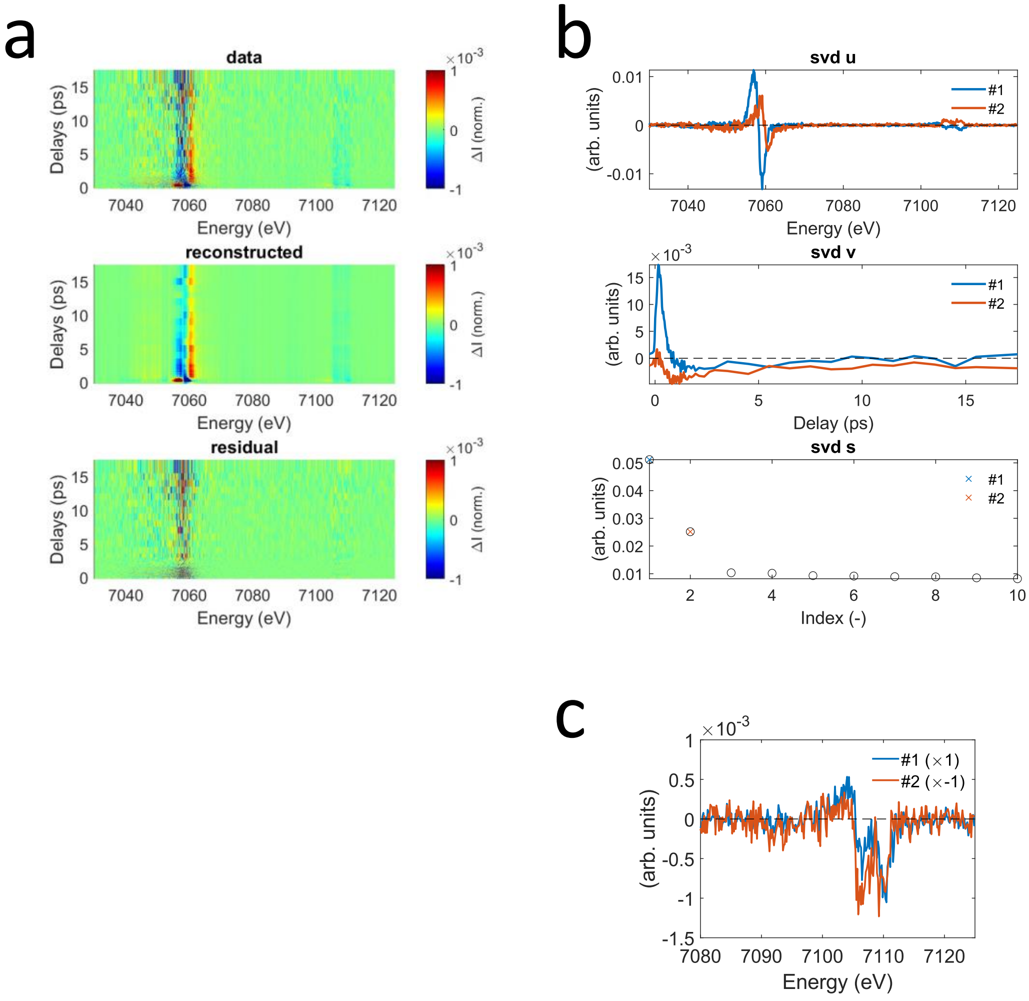

Supplementary Figure 3: Singular value decomposition of the time dependent XES difference map. (a) The top inset shows the data, the middle inset shows the reconstructed difference map using the first two components shown in (b) and the bottom inset shows the residual. (b) The top inset shows the first two spectral components, the middle inset shows the first two temporal components and the bottom inset shows the first ten singular values. (c) Comparison of the scaled first and second spectral components in the VtC range.

To calculate the time dependent first moment shift $\Delta\mu_{1,VtC}$ and intensity change ΔI_{VtC}, the time dependent VtC XES map in the 7090 – 7125 eV range was noise-filtered via singular value decomposition and data reconstruction using the first four components. $\Delta\mu_{1,VtC}$ and ΔI_{VtC} were then calculated based on the reconstructed VtC XES map $I_{VtC,rec}(E,t)$ and the residual of the reconstruction was utilized for an error estimate at each time and energy point. The time dependent first moment position is calculated using:

$$\mu_{1,VtC}(t_j) = \frac{\sum_i I_{VtC,rec}(E_i,t_j)\cdot E_i}{\sum_i I_{VtC,rec}(E_i,t_j)} \quad (2)$$

Here, the summation includes a 10 eV-wide window centered around 7108 eV. The error estimate from the time dependent VtC XES map was propagated accordingly. The same fit function (equation 1) as utilized for the populations N_{LMCT} and N_{S2} derived from the Kβ main line analysis was used to fit the first moment kinetics. Fit results are summarized in Supplementary Table 3. The fit curve resulting from model *C* is shown in Figure 3b.

	A	*B*	*C*
IRF FWHM (fs)	222 (fixed)	222 (fixed)	222 (fixed)
τ_1 (fs)	474 ± 99	358 ± 103	280 ± 115
τ_2 (ps)	-	-	9 ± 16
τ_3 (ps)	-	1000 (fixed)	-
R^2 (-)	0.876	0.886	0.890

Supplementary Table 3: Fit of the time dependent VtC first moment positions using equation 1 with up to two exponential decay constants. In all cases, the IRF FWHM parameter was fixed to 222 fs, the result from the Kβ main line analysis. *A*: Fitting with one exponential decay constant τ_1. *B*: Fitting with one exponential decay constant τ_1 and a long decay constant fixed to τ_3 = 1000 ps. *C*: Fitting with two decay constants τ_1 and τ_2.

The total VtC intensity was calculated by summing all data points in a spectral range around the $K\beta_{2,5}$ feature (7108 eV), and the error estimate for the XES map was propagated accordingly. The extracted time dependent curves are sensitive to the summation range, however for summation widths in the 5 - 10 eV range, no significant variation of the extracted curves was observed. The final time dependent total VtC intensity signal was therefore defined as the average signal from curves constructed using different widths in the 5 - 10 eV range. The variation between the individual curves was utilized to estimate a systematic contribution to the total uncertainty. The difference signal shown in Figure 3c was then calculated by subtracting the total VtC intensity of the $^2[Fe^{III}(CN)_6]^{3-}$ laser-off spectrum evaluated in the same way. The uncertainty in the laser-off spectrum was neglected to estimate the errors of the difference curve. Again, the same fit function (equation 1) as utilized for the populations N_{LMCT} and N_{S2} was used to fit ΔI_{VtC}. The fit results are summarized in Supplementary Table 4. Model *B* is shown in Figure 3c.

	A	*B*
IRF FWHM (fs)	222 (fixed)	222 (fixed)
τ_1 (ps)	-	-
τ_2 (ps)	5.1 ± 1.8	4.9 ± 2.8
τ_3 (ps)	-	1000 (fixed)
R^2 (-)	0.928	0.935

Supplementary Table 4: Fit of the time dependent total intensity change of the VtC x-ray emission difference signal using equation 1 with up to two exponential decay constants. In both cases, the IRF FWHM was fixed to 222 fs, the result from the Kβ main line analysis. *A*: Fitting with one exponential decay constant τ_2. *B*: Fitting with an exponential decay constant τ_2 and a second decay constant fixed to τ_3 = 1000 ps.

The DFT calculations are described in the Methods section of the main text and geometry-optimized structures and energies are summarized in Supplementary Tables 5 and 6. A comparison between calculated difference spectra and the experimental difference spectrum in the 1.5 – 3 ps range is shown in Supplementary Figure 4 together with the fitted scaling factors. Since all calculated spectra were normalized with the same factor determined from scaling the calculated $^2[Fe^{III}(CN)_6]^{3-}$ ground state spectrum to the background-subtracted experimental spectrum (Supplementary Figure 5), the fitted scaling factors shown in Figure 3d and Supplementary Figure 4 represent population fractions for these models. We have then utilized these population fractions to estimate the first moment shift that would be observed for each candidate species in the 1.5 – 3 ps range. The results are compared with the experimentally observed shift in Supplementary Figure 6.

	Symmetry	$(Fe-C)^{ax}$	$(Fe-C)^{eq}$	C-N	Fe-O	Energy
		(Å)	(Å)	(Å)	(Å)	(eV)
$^2[Fe^{III}(CN)_6]^{3-}$	O_h	1.96	1.96	1.16		0
$^4[Fe^{III}(CN)_6]^{3-}$	D_{4h}	2.29	2.00	1.16		1.19
$^6[Fe^{III}(CN)_6]^{3-}$	O_h	2.19	2.19	1.16		1.76
$^2[Fe^{III}(CN)_5]^{2-}$ (SP)	C_{4v}	1.88	1.95	1.16	-	1.72
$^2[Fe^{III}(CN)_5]^{2-}$ (TBP)	D_{3h}	1.94	1.88/1.98	1.16		1.96
$^4[Fe^{III}(CN)_5]^{2-}$ (SP)	C_{4v}	2.07	1.97	1.16	-	1.53
$^4[Fe^{III}(CN)_5]^{2-}$ (TBP)	D_{3h}	1.96	1.99/2.03	1.16	-	1.54
$^2[Fe^{III}(CN)_5H_2O]^{2-}$	C_{4v}/C_s	1.91	1.96	1.16	2.05	0.85

Supplementary Table 5: Approximate molecular symmetries, calculated bond lengths and energies for $^2[Fe^{III}(CN)_6]^{3-}$ and candidate photoproducts. To estimate relative energies with respect to the $^2[Fe^{III}(CN)_6]^{3-}$ ground state, energies for H_2O and CN^- were determined from separate DFT calculations.

Fe	-2.3E-06	2.32E-06	4.97E-06
C	1.958207	-0.0315	-0.0306
C	-1.95821	0.031527	0.030623
C	-0.03152	1.958283	-0.03183
C	0.031519	-1.95828	0.03184
C	-0.03274	-0.03181	1.958271
C	0.032742	0.0318	-1.95826
N	3.118824	-0.04289	-0.04633
N	-3.11883	0.042932	0.04631
N	-0.04291	3.118917	-0.04804
N	0.042914	-3.11891	0.048002
N	-0.04501	-0.04808	3.118864
N	0.045012	0.048013	-3.11886

Supplementary Table 6: Coordinates of DFT optimized ground state structure of $^{2}[Fe^{III}(CN)_6]^{3-}$.

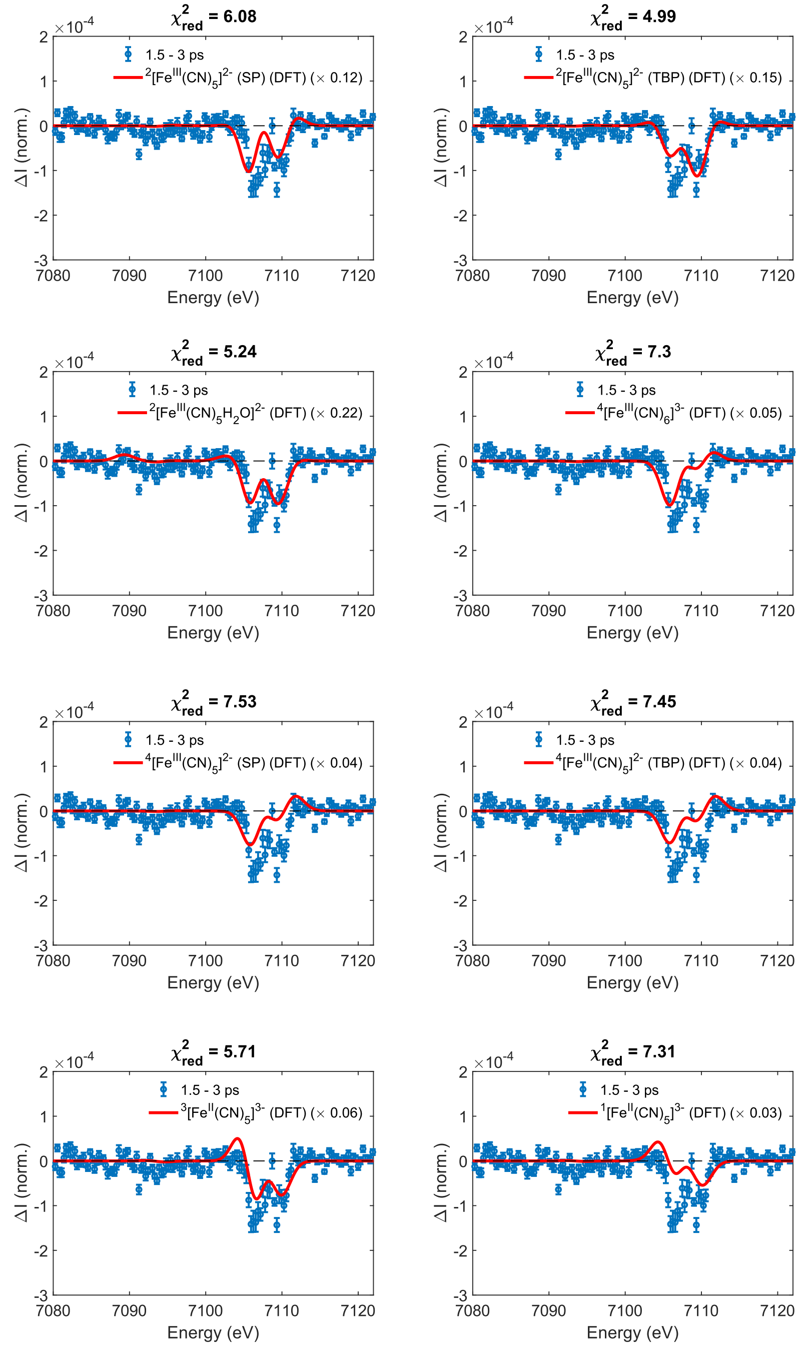

Supplementary Figure 4: The experimental valence-to-core XES difference spectrum averaged in the 1.5 – 3 ps range and spectrally binned is compared with calculated difference spectra for various candidate species. χ^2_{red} values are evaluated in the 7080 – 7122 eV range. Error bars were first estimated from the standard deviation within the time bin when all difference spectra are rescaled to the bin-averaged summed difference signal magnitude, then propagated for each spectral bin.

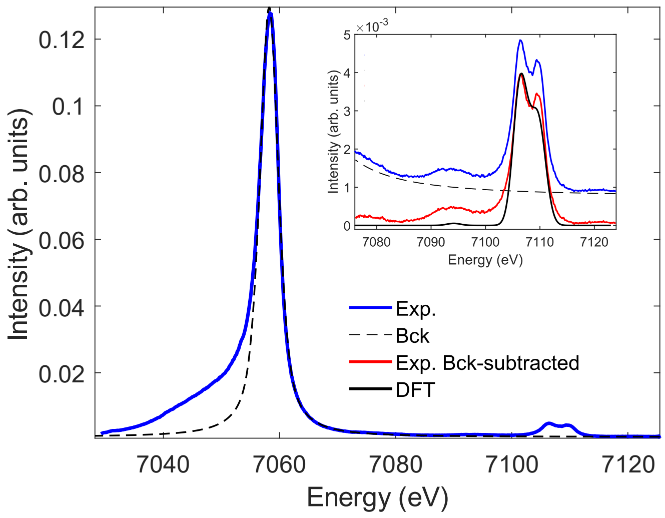

Supplementary Figure 5: The experimental $^{2}[Fe^{III}(CN)_6]^{3-}$ laser off spectrum is shown in blue. The black dashed line represents a fit using a single pseudo-Voigt function to subtract the background in the valence-to-core region. The inset shows the resulting background-subtracted experimental spectrum (red) and the scaled calculated $^{2}[Fe^{III}(CN)_6]^{3-}$ spectrum (black line).

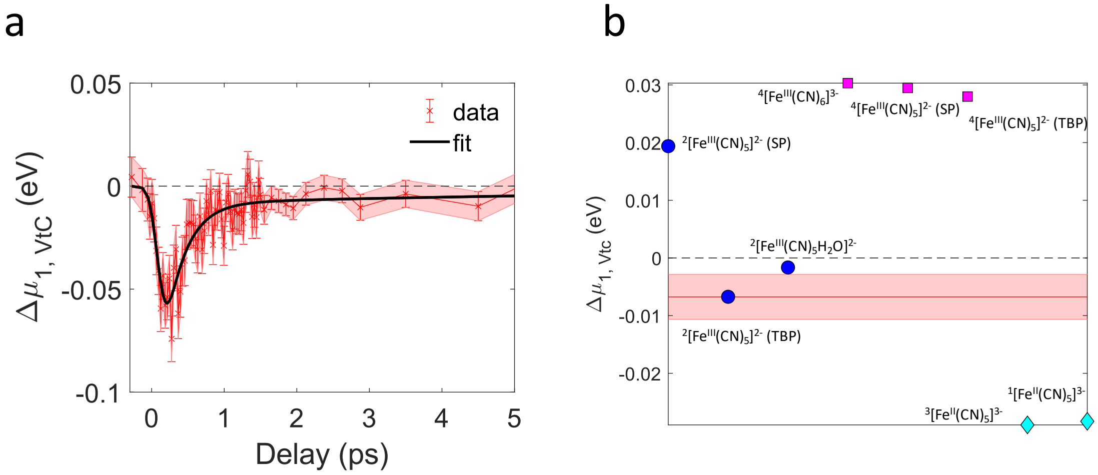

Supplementary Figure 6: (a) shows the time dependent first moment change in the VtC region from Figure 3b in a limited range up to 5 ps. (b) Shows the average first moment change in the 1.5 – 3 ps range (horizontal red line). The shaded area represents the uncertainty. Markers represent predicted first moment shifts for different candidate species, considering the calculated VtC spectra with fitted population fractions from Figure 3d / Supplementary Figure 4.

Supplementary Note 5: Analysis of the XSS difference signal in the 1.5 – 4.0 Å^{-1} range

Here, we analyze the 1.5 – 4.0 Å^{-1} range of the time dependent XSS difference signal (Figure 4a), which is sensitive to bulk water heating and density changes. No time dependent changes in shape are observed for this difference signal and a comparison with reference curves from Kjaer *et al.*[1] shows good agreement for the bulk water heating reference after applying a calibration shift. To extract the time dependent increase in bulk water temperature, we fit the XSS difference signal ΔS in the range Q = 1.5 – 4.0 Å^{-1} using the following equation:

$$\Delta S(Q,t) = \left.\frac{\partial S(Q)}{\partial T}\right|_{\rho} \cdot \Delta T(t) + \left.\frac{\partial S(Q)}{\partial \rho}\right|_{T} \cdot \Delta\rho(t) \qquad (3)$$

Here, we use the references from Kjaer *et al.*[1] for the difference scattering signals resulting from the change in bulk water temperature at constant density $\left.\frac{\partial S(Q)}{\partial T}\right|_{\rho}$ and the change in density at constant temperature $\left.\frac{\partial S(Q)}{\partial \rho}\right|_{T}$, respectively. From the fit, we find that the density increase $\Delta\rho$ is negligible (Supplementary Figure 7a). The increase in bulk water temperature ΔT (represented by the kinetic trace averaged in the 2.25 – 2.35 Å^{-1} shown in Supplementary Figure 7b) occurs due to excess energy dissipation from the solute that reaches a plateau after ~20 ps where the temperature increase is ~1.66 K. Using the specific heat at constant volume, $C_V \sim$74.54 J·mol^{-1}·K^{-1} and the number of water molecules per liquid unit cell $\frac{c_{H2O}}{c_{solute}}$, ΔT is converted into bulk water excess energy. We then assume that most of the excess energy deposited through the absorption of a 336 nm photon has dissipated into the bulk water and estimate the excitation fraction as f_{exc} = 0.19 ± 0.02, where the error estimate results from propagating a 10% error on c_{solute} and 0.02 K uncertainty on ΔT. This estimate constitutes a lower bound on the excitation fraction because it neglects any energy that is stored in long-lived photoproducts. To obtain an upper bound, we assume a 10% photo-aquation quantum yield.[2] The energy difference between $^2[Fe^{III}(CN)_6]^{3-}$ + H_2O and $^2[Fe^{III}(CN)_5H_2O]^{2-}$ + CN^- estimated from DFT (Supplementary Table 5) is ~0.85 eV. The measured bulk water temperature increase of 1.66 K then translates into an excitation fraction of f_{exc} = 0.20 ± 0.02.

The kinetic fit of the temperature evolution (Supplementary Figure 7b) requires two exponential time constants fitted to ~1.3 ps and ~5.8 ps, respectively. Similar time constants for solute-solvent excess energy dissipation were found by Ojeda *et al.* from their transient IR measurements and assigned to the relaxation of photoexcited low- and high-frequency vibrational modes.[3] The similarity of these time constants with the 1 - 5 ps partial lifetime of the proposed ligand photolysis intermediates (determined from XES and the XSS low-Q difference signal shown in Supplementary Figure 7b) may also suggest a correlation between the hexa- and penta-coordinate populations and the amount of solute vibrational excess energy.[4] Higher amounts of excess energy should favor the dissociated complexes, thus preventing rapid rebinding of a cyanide anion or attachment of a water molecule. While we are unable to

experimentally distinguish between different geometries of the penta-coordinate complex, cyanide dissociation most likely forms an SP geometry before interconversion with the TBP form occurs.[5] Net conversion towards the TBP geometry, if occurring, should then effectively decrease the rate at which the hexa-coordinate $^2[Fe^{III}(CN)_6]^{3-}$ and $^2[Fe^{III}(CN)_5H_2O]^{2-}$ complexes can form, due to steric constraints. While spectroscopically, we cannot reliably distinguish the penta-coordinate and aquated complexes, the persistent offset in the Kβ main line XES differences is consistent with the formation of the long-lived $^2[Fe^{III}(CN)_5H_2O]^{2-}$ complex.

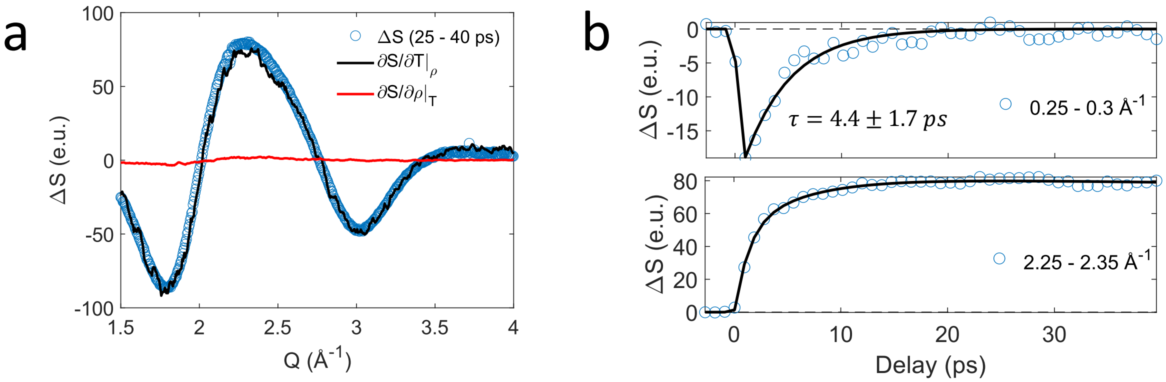

Supplementary Figure 7: (a) Average of the time dependent XSS difference signal (shown in Figure 4a) in the 25 – 40 ps range. The fitted bulk water heat and density differentials are shown in black and red, respectively. (b) Time dependence of the low-Q difference signal (upper inset) and on the maximum of the water heating difference signal (lower inset).

Supplementary Note 6: XES global target analysis and species associated difference spectra

Leveraging the combined Fe Kβ main line and VtC spectral range of the time dependent XES data, we have also extracted species associated difference spectra (SADS). We use the kinetic scheme for the photo-aquation reaction shown in Figure 5a and Supplementary Figure 8a. Here we neglect spectral differences between the SP and TBP geometries of the penta-coordinate complex. Specifically, we have solved the following rate equations:

$$\dot{N}_{GS}(t) = k_{rec} \cdot N_{penta}(t) \qquad (4a)$$

$$\dot{N}_{LMCT}(t) = -k_{LMCT} \cdot N_{LMCT}(t) \qquad (4b)$$

$$\dot{N}_{MC}(t) = k_{LMCT} \cdot N_{LMCT}(t) - k_{MC} \cdot N_{MC}(t) \qquad (4c)$$

$$\dot{N}_{penta}(t) = k_{MC} \cdot N_{MC}(t) - (k_{rec} + k_{aqua}) \cdot N_{penta}(t) \qquad (4d)$$

$$\dot{N}_{aqua}(t) = k_{aqua} \cdot N_{penta}(t) \qquad (4e)$$

The initial conditions were chosen as $N_{GS}(0) = 1 - f_{exc}$, $N_{LMCT}(0) = f_{exc}$, $N_{MC}(0) = N_{penta}(0) = N_{aqua}(0) = 0$. From the XSS analysis (Supplementary Note 5), we then use $f_{exc} = 0.20$. To determine $\tau_{LMCT} = {k_{LMCT}}^{-1}$ and $\tau_{penta} = (k_{rec} + k_{aqua})^{-1}$, we performed a global fit of the time dependent XES data in the combined Fe Kβ main line and VtC range using equation 1 (Supplementary Figure 8b). The third time constant was fixed to τ_3 = 1000 ps to account for the persistent component in the XES data. The global fit was performed using the first two SVD temporal components (Supplementary Figure 3) with common time constants IRF FWHM, t_0, τ_1 - τ_3 and independent coefficients a_1 - a_3. The IRF FWHM was fixed to 222 fs (Supplementary Note 3) and k_{MC} is set to a large value to avoid any population build-up in the MC ES. We then find a faster time constant $\tau_1 = \tau_{LMCT} \approx$ 275 fs and a slower time constant $\tau_2 = \tau_{penta} \approx$ 3.5 ps, consistent with the time constants reported from the separate analysis of the Fe Kβ main line (Supplementary Note 3) and VtC (Supplementary Note 4) regions.

Furthermore, we use $k_{rec} = \frac{1-\phi_{aqua}}{\tau_{penta}}$ and $k_{aqua} = \frac{\phi_{aqua}}{\tau_{penta}}$. Consistency of our model with quantum yields reported by Fuller *et al.*[2] requires $\phi_{aqua} = 0.02 - 0.06$. Importantly, only $N_{aqua}(t)$ depends on the choice of ϕ_{aqua} while the extracted populations $N_{LMCT}(t)$ and $N_{penta}(t)$ are independent from ϕ_{aqua}. We therefore fix $\phi_{aqua} = 0.05$ in our model.

We then use the extracted time dependent populations $N_{LMCT}(t)$, $N_{penta}(t)$ and $N_{aqua}(t)$ shown in Supplementary Figure 8c to solve the following overdetermined system of equations at each energy point E_i:

$$\Delta I_{XES,rec}(E_i, t_j) = \sum_k N_k(t_j) \cdot \Delta I_k(E_i) \qquad (5)$$

Here, $\Delta I_{XES,rec}$ is the noise-filtered, reconstructed data using two components and $k = LMCT, penta, aqua$. This yields the SADS ΔI_k shown in Figure 5b for the $^2T_{2u}$ LMCT ES and penta-coordinate intermediate, and additionally for $^2[Fe^{III}(CN)_5H_2O]^{2-}$ in Supplementary Figure 8d. Again, the SADS for the LMCT ES, and penta-coordinate complex do not depend on ϕ_{aqua} while for $^2[Fe^{III}(CN)_5H_2O]^{2-}$, the ΔI_{aqua} extracted for different choices of ϕ_{aqua} are identical up to a scaling factor.

ΔI_{LMCT} agrees well with the experimental LMCT ES model differences shown in Figures 2a and 3a for the Fe Kβ main line and VtC spectral ranges, respectively. In Supplementary Figure 8e, we also compare ΔI_{LMCT} with the DFT-based difference of the $^1[Fe^{II}(CN)_6]^{4-}$ and $^2[Fe^{III}(CN)_6]^{3-}$ VtC spectra.

ΔI_{penta} exhibits a blueshift in the Fe Kβ main line region consistent with the calculated $K\beta_{1,3}$ peak shift between $^2[Fe^{III}(CN)_5]^{2-}$ and $^2[Fe^{III}(CN)_6]^{3-}$ shown in Figure 2d. In the VtC region, ΔI_{penta} exhibits a reduction in intensity while positive features are absent. Again, this observation is fully consistent with the calculated VtC spectra for the proposed penta-coordinate doublet intermediates (Supplementary Figure 8f). Supplementary Figure 8f also shows that the magnitude of ΔI_{penta} is similar to that of the difference signals calculated for the penta-coordinate doublet intermediates while the hexa- and penta-coordinate quartet complexes exhibit larger difference signal magnitudes.

For ΔI_{aqua}, the achieved signal-to-noise of the XES data is insufficient to allow for a robust analysis. The SVD of the XES data does not capture the subtle spectral evolution that potentially occurs after ~1.5 ps as suggested in Supplementary Note 1. Therefore, the main difference between ΔI_{aqua} and ΔI_{penta} is a scaling factor while the two SADS have practically the same spectral shape (Supplementary Figures 8d, 8f and 8g).

The larger magnitude of the $K\beta_{1,3}$ peak shift for ΔI_{aqua} compared with ΔI_{penta} results from the small aquation yield ϕ_{aqua} assumed in our model and is qualitatively consistent with the calculated $K\beta_{1,3}$ peak shifts for $^2[Fe^{III}(CN)_5]^{2-}$ and $^2[Fe^{III}(CN)_5H_2O]^{2-}$ with respect to $^2[Fe^{III}(CN)_6]^{3-}$ (Figure 2d).

In summary, the extracted SADS in the combined Fe Kβ and VtC spectral range are consistent with the calculated spectra for the photo-aquation reaction intermediates and product (Figures 2d and 3d), thus further supporting the proposed photoinduced reaction scheme (Figure 5a).

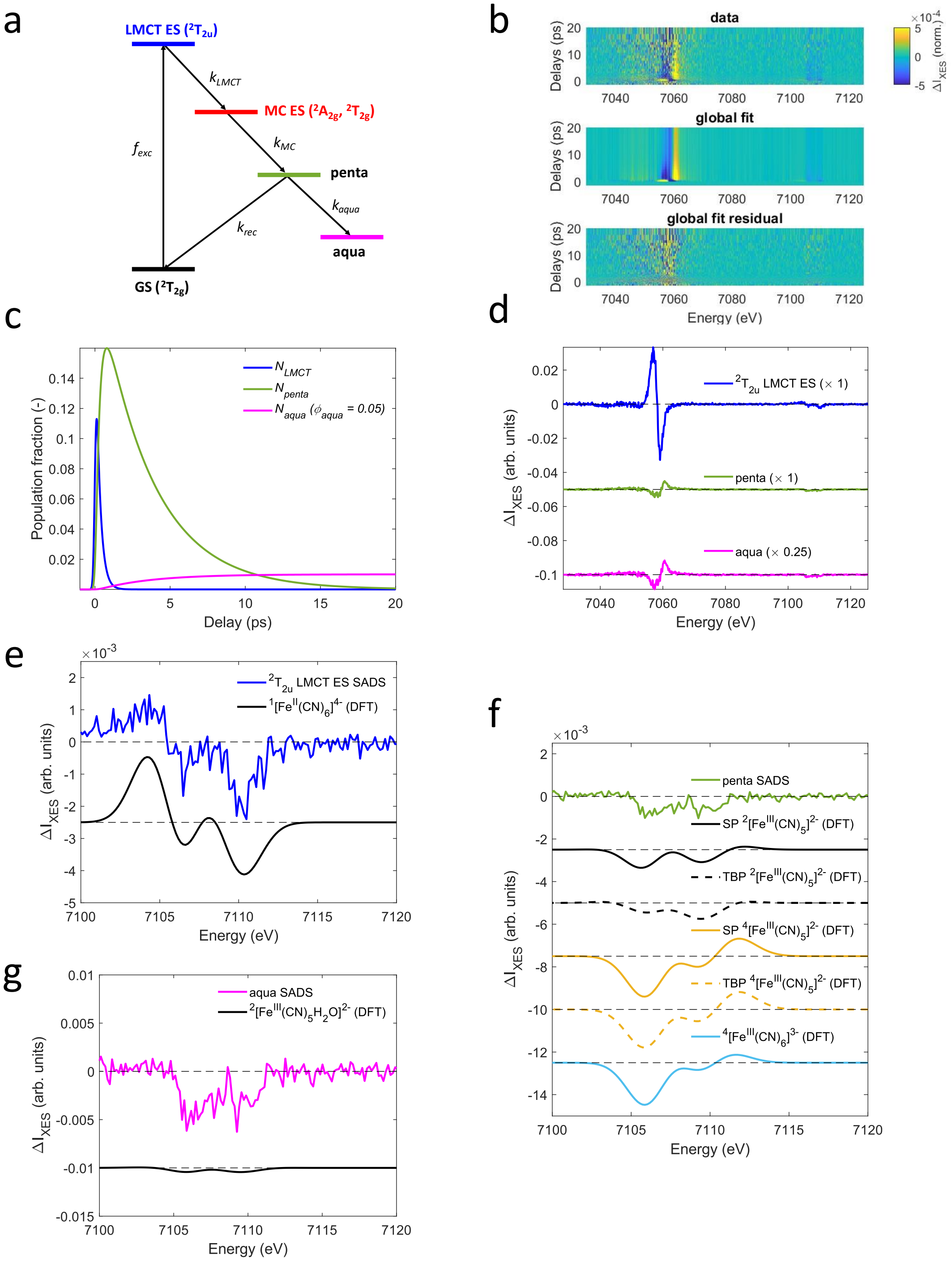

Supplementary Figure 8: (a) The kinetic scheme shows the species proposed to be involved in the photoinduced dynamics of aqueous $^{2}[Fe^{III}(CN)_6]^{3-}$ when excited at 336 nm. Symmetry labels are shown for the $^{2}[Fe^{III}(CN)_6]^{3-}$ ground and excited states. (b) The top inset shows the time dependent XES data in the combined Fe Kβ main line and VtC range up to 20 ps. The middle inset shows the map constructed by using the first two spectral SVD components and a global fit of

the first two temporal SVD components. The bottom inset shows the residual. (c) Time dependent populations calculated for species proposed in the kinetic scheme. (d) The SADS are shown for the $^{2}T_{2u}$ LMCT ES, penta-coordinate and aquated ($^{2}[Fe^{III}(CN)_5H_2O]^{2-}$) species. (e) Comparison of the $^{2}T_{2u}$ LMCT ES SADS with the DFT-based difference spectrum of $^{1}[Fe^{II}(CN)_6]^{4-}$ and $^{2}[Fe^{III}(CN)_6]^{3-}$ in the VtC region. (f) Comparison of the SADS for the penta-coordinate intermediate with DFT-based difference spectra for SP / TBP $^{2}[Fe^{III}(CN)_5]^{2-}$ / $^{4}[Fe^{III}(CN)_5]^{2-}$ and $^{4}[Fe^{III}(CN)_6]^{3-}$ in the VtC region. The DFT-based difference spectra are also shown in Figure 3d (scaled). (g) Comparison of the SADS for $^{2}[Fe^{III}(CN)_5H_2O]^{2-}$ with the DFT-based difference spectrum in the VtC region. The DFT-based difference spectrum is also shown in Figure 3d (scaled).

Supplementary Note 7: Calculation of the solute-solute XSS difference signal

The time dependent XSS difference signal arises from changes in solute-solute, solute-solvent, and solvent-solvent atom pair distances and can therefore be expressed as $\Delta S(Q,t) = \Delta S_{solute-solute}(Q,t) + \Delta S_{solute-solvent}(Q,t) + \Delta S_{solvent-solvent}(Q,t)$.[6] Supplementary Figure 9 shows calculations of $\Delta S_{solute-solute}$ using the Debye scattering equation[7] with coordinates for the optimized SP $^2[Fe^{III}(CN)_5]^{2-}$ (with a dissociated cyanide placed 2.4 Å away from the Fe-center) and $^2[Fe^{III}(CN)_5H_2O]^{2-}$ structures determined from DFT and reported in Supplementary Table 5. Difference scattering signals were calculated by subtracting the calculated $^2[Fe^{III}(CN)_6]^{3-}$ XSS signal. The difference scattering signals were scaled considering average populations of the penta-coordinate and aquated complexes determined from the populations shown in Supplementary Figure 8c. The black lines in Supplementary Figure 9 represent the sum of these two contributions. Importantly, as these calculated XSS differences only represent the contribution of the $\Delta S_{solute-solute}$ contribution to the total XSS difference signal while the solute-solvent cross term and solvent contribution are completely ignored, agreement with the observed difference signal is not expected. In particular, the disagreement between the calculated and measured difference scattering signal in the low-Q region may be due to the absence of the $\Delta S_{solute-solvent}$ term. The calculation of this contribution requires extensive molecular dynamics simulations which is outside the scope of this work.

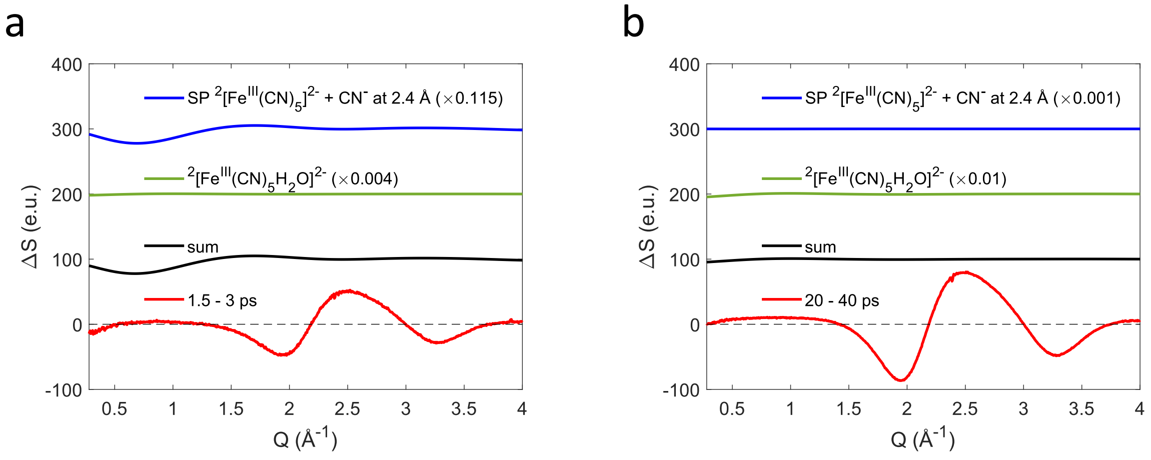

Supplementary Figure 9: Simulated XSS difference signals for isolated SP $^2[Fe^{III}(CN)_5]^{2-}$ and $^2[Fe^{III}(CN)_5H_2O]^{2-}$ solute structures from Supplementary Table 5. Scaling factors represent average populations determined from the populations shown in Supplementary Figure 8c. The black line represents the sum of the SP $^2[Fe^{III}(CN)_5]^{2-}$ and $^2[Fe^{III}(CN)_5H_2O]^{2-}$ contributions and the red line represents the experimental difference spectrum averaged in the same time range. (a) and (b) show two different time ranges.

Supplementary Note 8: Dependence of the VtC x-ray emission spectrum on the incident x-ray energy

The time resolved x-ray emission spectra have been measured using an incident x-ray energy of 8.5 keV. To assess whether at this energy, there are significant contributions of multivacancy satellite features in the VtC region, we have compared our $^2[Fe^{III}(CN)_6]^{3-}$ laser off spectrum with previously reported spectra measured for the same compound, but using different incident x-ray energies (Supplementary Figure 10). Ross *et al.*[8] have used an incident x-ray energy of 7.5 keV, therefore KβL satellites should not have contributed to their spectra. Based on the good agreement of our spectrum in the range 7103 – 7116 eV where the relevant transient features are observed in the difference data, we conclude that our spectra are not significantly affected by satellite lines that could complicate the data analysis.

We have also compared our $^2[Fe^{III}(CN)_6]^{3-}$ spectrum with that measured by Lee *et al.*[9] using an incident x-ray energy of 9 keV.

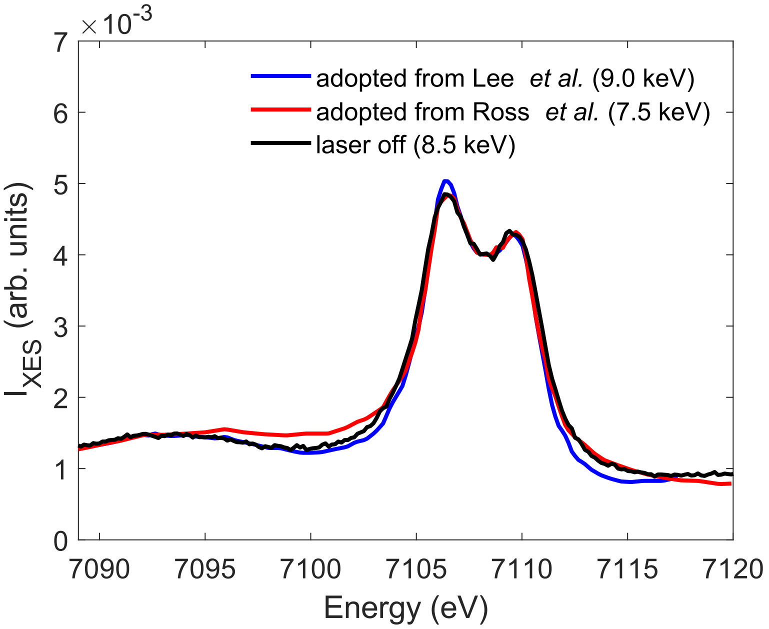

Supplementary Figure 10: Comparison between the measured $^2[Fe^{III}(CN)_6]^{3-}$ laser off spectrum (black line) in the VtC range and reference $^2[Fe^{III}(CN)_6]^{3-}$ spectra taken from Ross *et al.*[8] and Lee *et al.*[9] Both reference spectra were manually digitized from their manuscript figures and interpolated to the same energy axis of our measurement, applying a common energy shift of 0.31 eV. A constant background subtraction and scaling factor were applied to these spectra for comparison.

References

1. Kjaer, K. S.; van Driel, T. B.; Kehres, J.; Haldrup, K.; Khakhulin, D.; Bechgaard, K.; Cammarata, M.; Wulff, M.; Sorensen, T. J.; Nielsen, M. M., Introducing a standard method for experimental determination of the solvent response in laser pump, X-ray probe time-resolved wide-angle X-ray scattering experiments on systems in solution. *Physical Chemistry Chemical Physics* **2013,** *15* (36), 15003-15016.
2. Fuller, M.; Lebrocq, K.; Leslie, E.; Wilson, I., The Photolysis of Aqueous-Solutions of Potassium Hexacyanoferrate(III). *Australian Journal of Chemistry* **1986,** *39* (9), 1411-1419.
3. Ojeda, J.; Arrell, C. A.; Longetti, L.; Chergui, M.; Helbing, J., Charge-transfer and impulsive electronic-to-vibrational energy conversion in ferricyanide: ultrafast photoelectron and transient infrared studies. *Physical Chemistry Chemical Physics* **2017,** *19* (26), 17052-17062.
4. Mara, M. W.; Hadt, R. G.; Reinhard, M. E.; Kroll, T.; Lim, H.; Hartsock, R. W.; Alonso-Mori, R.; Chollet, M.; Glownia, J. M.; Nelson, S.; Sokaras, D.; Kunnus, K.; Hodgson, K. O.; Hedman, B.; Bergmann, U.; Gaffney, K. J.; Solomon, E. I., Metalloprotein entatic control of ligand-metal bonds quantified by ultrafast x-ray spectroscopy. *Science* **2017,** *356* (6344), 1276.
5. March, A. M.; Doumy, G.; Andersen, A.; Haddad, A. A.; Kumagai, Y.; Tu, M.-F.; Bang, J.; Bostedt, C.; Uhlig, J.; Nascimento, D. R.; Assefa, T. A.; Németh, Z.; Vankó, G.; Gawelda, W.; Govind, N.; Young, L., Elucidation of the photoaquation reaction mechanism in ferrous hexacyanide using synchrotron x-rays with sub-pulse-duration sensitivity. *The Journal of Chemical Physics* **2019,** *151* (14), 144306.
6. Biasin, E.; Fox, Z. W.; Andersen, A.; Ledbetter, K.; Kjær, K. S.; Alonso-Mori, R.; Carlstad, J. M.; Chollet, M.; Gaynor, J. D.; Glownia, J. M.; Hong, K.; Kroll, T.; Lee, J. H.; Liekhus-Schmaltz, C.; Reinhard, M.; Sokaras, D.; Zhang, Y.; Doumy, G.; March, A. M.; Southworth, S. H.; Mukamel, S.; Gaffney, K. J.; Schoenlein, R. W.; Govind, N.; Cordones, A. A.; Khalil, M., Direct observation of coherent femtosecond solvent reorganization coupled to intramolecular electron transfer. *Nature Chemistry* **2021,** *13* (4), 343-349.
7. Christensen, M.; Haldrup, K.; Bechgaard, K.; Feidenhans'l, R.; Kong, Q.; Cammarata, M.; Russo, M. L.; Wulff, M.; Harrit, N.; Nielsen, M. M., Time-Resolved X-ray Scattering of an Electronically Excited State in Solution. Structure of the 3A2u State of Tetrakis-μ-pyrophosphitodiplatinate(II). *Journal of the American Chemical Society* **2009,** *131* (2), 502-508.
8. Ross, M.; Andersen, A.; Fox, Z. W.; Zhang, Y.; Hong, K.; Lee, J.-H.; Cordones, A.; March, A. M.; Doumy, G.; Southworth, S. H.; Marcus, M. A.; Schoenlein, R. W.; Mukamel, S.; Govind, N.; Khalil, M., Comprehensive Experimental and Computational Spectroscopic Study of Hexacyanoferrate Complexes in Water: From Infrared to X-ray Wavelengths. *The Journal of Physical Chemistry B* **2018,** *122* (19), 5075-5086.
9. Lee, N.; Petrenko, T.; Bergmann, U.; Neese, F.; DeBeer, S., Probing Valence Orbital Composition with Iron Kβ X-ray Emission Spectroscopy. *Journal of the American Chemical Society* **2010,** *132* (28), 9715-9727.
